# Relation between Serum Creatine Phosphokinase Levels and Acute Kidney Injury among ST-Segment Elevation Myocardial Infarction Patients

**DOI:** 10.3390/jcm11041137

**Published:** 2022-02-21

**Authors:** David Zahler, Keren-Lee Rozenfeld, Ilan Merdler, Tamar Itach, Samuel Morgan, Dana Levit, Shmuel Banai, Yacov Shacham

**Affiliations:** 1 Department of Cardiology, Tel-Aviv Sourasky Medical Center Affiliated to the Sackler Faculty of Medicine, Tel-Aviv University, Tel-Aviv 64239, Israel; kerenlees@gmail.com (K.-L.R.); ilanmerdler@gmail.com (I.M.); samuelmorgan@mail.tau.ac.il (S.M.); shmuelb@tlvmc.gov.il (S.B.); kobys@tlvmc.gov.il (Y.S.); 2 Internal Medicine Department H, Tel-Aviv Sourasky Medical Center Affiliated to the Sackler Faculty of Medicine, Tel-Aviv University, Tel-Aviv 64239, Israel; itachtamar@gmail.com (T.I.); levit.dana@gmail.com (D.L.)

**Keywords:** ST-segment elevation myocardial infarction, acute myocardial infarction, creatine phosphokinase, acute kidney injury

## Abstract

Background: Among patients with rhabdomyolysis, the leakage of intracellular skeletal muscle content such as creatine phosphokinase (CPK) into the bloodstream has been associated with an increased risk of acute kidney injury (AKI). We evaluated the possible relationship between serum CPK levels and AKI occurrence among patients with myocyte injury secondary to ST-elevation myocardial infarction (STEMI). Methods: We retrospectively included 2794 patients with STEMI. Patients were stratified according to peak serum CPK levels into mild (<1000 U/L, *n* = 1603), moderate (1000–5000 U/L, *n* = 1111), and severe (>5000 U/L, *n* = 80) categories. The occurrence of AKI was defined by the KDIGO criteria as an increase in serum creatinine (sCR) ≥0.3 mg/dL within 48 h following PCI. The predictive value of CPK for the risk of AKI occurrence was assessed using multivariate logistic regression models. Results: The overall occurrence of AKI was 10.4%. Incidence of AKI showed a gradual increase between patients with mild, moderate, and severe serum CPK level elevations (7.8% vs. 11% vs. 26% respectively; *p* < 0.001). In multivariate logistic regression models, both moderate or higher (OR 1.6, 95% CI 1.1–2.2; *p* = 0.01) and severe (OR 2.8 95% CI 1.4–5.6; *p* = 0.004) serum CPK level elevations were independently associated with AKI. Conclusions: Among STEMI patients, elevated CPK levels were associated with AKI. This association is presumably independent; however, it remains unclear whether it is due to direct toxic (myoglobin-related) or hemodynamic effects (poor left ventricular function). Further studies are required to reveal the underlying mechanism.

## 1. Introduction

Severe rhabdomyolysis (RM) is often complicated by acute kidney injury (AKI) [1]. Muscular cell wall disruption causes the release of intracellular elements such as myoglobin and creatine phosphokinase (CPK) [2]. Although not directly implicated in the pathogenesis of AKI during RM, CPK is routinely used as a serum biomarker for skeletal muscle damage and correlates with the risk of renal injury [3]. Creatine phosphokinase myocardial band (CPK-MB), an isoenzyme of CPK, is a well-established marker of myocardial cell damage [4]. At the time of acute myocardial infarction (AMI), increases in both CPK-MB and total CPK levels are observed [5].

AKI following AMI is common and associated with adverse outcomes [6,7]. However, as opposed to skeletal muscle disruption, the possible predictive significance of CPK as a marker for impending AKI in acute myocardial damage has not yet been investigated. We aimed to evaluate the possible utility of CPK as a marker for AKI occurrence among ST-elevation myocardial infarction (STEMI) patients.

## 2. Material and Methods

We performed a retrospective, single-center observational study at the Tel-Aviv Sourasky Medical Center, a tertiary referral hospital with a 24/7 primary PCI service. We included 2958 consecutive patients admitted between October 2007 and December 2019 to the cardiac intensive care unit (CICU) with a diagnosis of acute STEMI. Patients treated either conservatively or by thrombolysis were excluded (*n* = 31), as well as 63 patients whose diagnosis on discharge was a condition other than STEMI (e.g., myocarditis or takotsubo cardiomyopathy). In addition, we excluded patients who died within 24 h of admission (*n* = 45), presuming insufficient time for AKI to occur. Patients requiring chronic dialysis treatment (*n* = 12) or patients with no documented serum CPK levels (*n* = 13) were excluded as well.

The final study population included 2794 patients whose baseline demographics, cardiovascular history, clinical risk factors, treatment characteristics, and laboratory results were all retrieved from hospital electronic medical records. Diagnosis of STEMI was established in accordance with published guidelines including a typical chest pain history, diagnostic electrocardiographic changes, and serial elevation of cardiac biomarkers [8]. Coronary angioplasty was performed on patients with symptoms ≤ 12 h in duration as well as in patients with symptoms lasting 12–24 h in duration if the symptoms persisted at the time of admission.

### 2.1. Laboratory Data

Serum CPK levels were measured upon CICU admission and at least once daily until discharge. Peak CPK levels were defined as the highest level obtained throughout hospitalization. Although there is no existing unified definition, patients were stratified into three groups based on peak serum CPK levels: mild (<1000 U/L), moderate (1000–5000 U/L), and severe (>5000 U/L), as suggested in previous reports [9,10,11].

The serum creatinine (sCr) level was determined at hospital admission, prior to primary PCI and at least daily throughout the CICU stay, and was available for all analyzed patients. Estimated glomerular filtration rate (eGFR) was determined using the Chronic Kidney Disease Epidemiology Collaboration (CKD-EPI) formula [12].

Admission eGFR of ≤60 mL/min/1.73 m^2^ defined patients with chronic kidney disease (CKD) [13]. AKI was diagnosed by the Kidney Disease Improving Global Outcomes (KDIGO) criteria and defined as an sCr rise ≥0.3 mg/dL within 48 h following CICU admission, compared with admission levels [13]. In a subset of 224 patients admitted to the CICU between February 2019 and December 2019, venous blood was drawn 24 h following CICU admission in order to evaluate plasma neutrophil gelatinase-associated lipocalin (NGAL) levels. NGAL levels were analyzed using NGAL rapid ELISA kits (Bioporto Diagnostics, Copenhagen, Denmark).

### 2.2. Statistical Analysis

Continuous variables were presented as mean and standard deviation and compared with one-way analysis of variance (ANOVA) when normally distributed. Median and interquartile range were used in non-normally distributed variables. These variables were compared with the Kruskal–Wallis H test. Categorical variables are presented as percentages, and *p* values were calculated with the chi-square test. Independent predictors of AKI were determined using multivariate binary logistic regression models adjusted for all baseline variables found to be significant in univariate analysis. A two-tailed *p* value of <0.05 was considered significant for all analyses. All analyses were performed with the SPSS software (SPSS Inc., Chicago, IL, USA).

## 3. Results

A total of 2794 patients were included (81% males, mean age of 62 ± 13), of whom 1603 (57%) had mild, 1111 (40%) had moderate, and 80 (3%) had severe peak CPK level elevations. The median time for peak CPK measurement was 19 (IQR 12–28) hours. Baseline demographic and clinical characteristics for the study population stratified by CPK levels are presented in Table 1. Patients with higher CPK levels were more likely to be younger men and to have fewer comorbidities. In addition, patients with higher CPK levels showed lower left ventricular ejection fraction and higher peak troponin levels.

**Figure 1:**
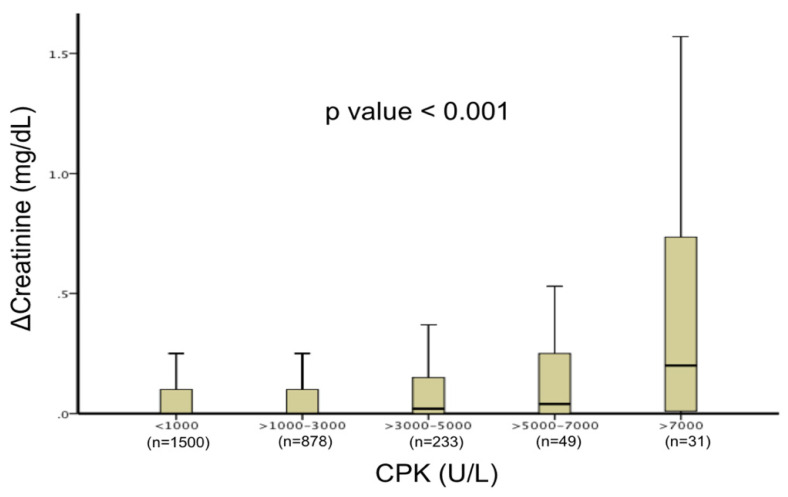
Relationship between CPK level elevations and creatinine changes. Box plot chart demonstrating creatinine changes stratified by peak creatine phosphokinase levels during hospitalization. A gradual increase in creatinine change was observed with rising CPK levels (*p* for trend <0.001).

Table 2 presents the kidney-related baseline and outcome data. Patients with higher CPK level elevations were more likely to have chronic kidney disease. There was a gradual increase in the occurrence of AKI among the CPK groups (mild 7.8%, moderate 11%, severe 26%, *p* < 0.001). Similar increases were observed in sCr change, peak sCr, and 24 h plasma NGAL levels (in a subset of 224 patients in whom NGAL levels were available). More significant CPK level elevations correlated with rising sCr (*p* < 0.001, Figure 1). In two multivariate binary logistic regression models including all variables found to be predictive in univariate analysis, CPK levels were independently associated with a higher risk of AKI (CPK ≥ 1000 (OR 1.6, 95% CI 1.1–2.2; *p* = 0.01) and CPK ≥ 5000 (OR 2.8, 95% CI 1.4–5.6; *p* = 0.004), Table 3).

## 4. Discussion

To our knowledge, the present study is the first to describe an independent predictive value for CPK levels regarding the risk of AKI in STEMI patients, and indicates a presumable additional mechanism for kidney failure in AMI patients.

While a similar linkage is well-described for skeletal muscle breakdown [14], no study has evaluated this possible association in acute myocardial cell injury to date.

CPK contains dimers of M and B chains, resulting in three different forms of isoenzymes [15]. CPK-MM is generally found in high amounts in skeletal muscle cells, whereas myocardial cells consist mostly of the CK-MB fraction [4]. Although CK-MB is no longer routinely recommended for diagnosis of AMI [16], total CPK is often measured at patient admission as part of a routine chemistry blood panel. CPK is also the standard laboratory biomarker for the diagnosis and severity assessment of skeletal muscle breakdown in RM, and correlates strongly with AKI risk [1]. Myocardial cell destruction as in the setting of STEMI mostly causes concomitant elevation of CPK-MB as well as total CPK [5,17].

Rhabdomyolysis is accompanied by renal impairment in up to 45% of patients and AKI is considered its most serious complication [14,18]. Concomitant renal injury may aggravate electrolyte abnormalities and is associated with increased mortality [3,19]. Myoglobinuria is the cause for renal damage in this setting [20] caused by direct myoglobin toxicity to renal tubular cells, obstructive intra-tubular casts, and myoglobin-mediated renal vasoconstriction [1].

However, myoglobin has a very short half-time in serum of only a couple of minutes and is therefore seldom measured in suspected RM or myocardial infarction [21]. Furthermore, studies focusing on measuring myoglobin levels failed to establish a clear correlation to the risk of renal injury [22,23].

Elevated CPK levels were repeatedly associated with acute renal failure and the need for renal replacement therapy in patients with RM [19,24]. While there are no clearly defined threshold values for increased risk [14], the incidence of AKI was significantly elevated with values above 15,000 [3,19]. Nevertheless, much lower values have been proposed as well [24], especially with other factors concomitantly affecting kidney function [14,25].

AKI is a frequent complication in STEMI and is associated with increased mortality [6,7]. The worsening of renal function is multifactorial. Besides the widely known deleterious impact of contrast volume [26], other contributing factors have been shown to be involved as well. Hemodynamic changes causing impaired cardiac output, vasoactive neurohormonal activation, hypoxemia, inflammation, acidosis, hyperglycemia, and administrated nephrotoxic drugs have all been shown to correlate with worsening renal function in AMI patients [27,28].

To date, the direct impact of spillage of myocardial cell content on renal function has not been demonstrated. Although this pathogenic mechanism is well-described in skeletal muscle breakdown, with CPK serving as a sensitive independent prognostic biomarker for impending renal dysfunction, this has not yet been tested in STEMI causing cardiac muscle destruction. We observed an increased risk of kidney dysfunction even at relatively low CPK level elevations, with risk starting to rise at moderate elevations (>1000–5000 U/L). This might be explained by the influence of the various deleterious mechanisms mentioned above, affecting the kidney simultaneously during AMI [27,28].

In the current cohort, we observed stepwise plasma NGAL level elevations among the different CPK groups. Recent data suggest the utilization of biomarkers of renal tubular damage (e.g., NGAL) in STEMI patients. These biomarkers may identify structural renal damage associated with adverse outcomes, even without functional AKI and preceding sCR elevation, thus serving as a useful tool for early AKI detection. However, they do not allow discrimination between toxic (myoglobin related) and ischemic (hemodynamic related) tubular injury [29,30].

Interestingly, the presence of hypertension was associated with an increased risk of AKI. While previous data do not point to an independent direct relation between baseline hypertension and AKI, we believe that this finding in the present study may be explained by other obscure factors not included in our models associated with hypertension increasing the risk of AKI in patients receiving contrast media.

### 4.1. Study Limitations

As a single-center, retrospective, non-randomized observational study, the cohort may have been subject to bias, even though we included consecutive patients and attempted to adjust for potential confounding factors using the multivariate regression model. The risk of AKI was strongly associated with baseline eGFR and LV function. Lower eGFR and LVEF were the two strongest predictors for AKI in our study, which were stronger than peak CPK levels. Furthermore, the number of patients with CPK levels over 5000 was small. These patients had lower eGFR at baseline and lower LVEF, so it remains unclear whether the effect on kidney function was solely due to higher CPK levels.

Data regarding the concomitant use of potentially nephrotoxic drugs such as angiotensin-converting enzyme inhibitors, mineralocorticoid receptor blockers, or diuretics was not present for many patients. Thus, we could not assess their effect on AKI development. In a similar sense, effects on CPK levels secondary to statin administration were not accessible for evaluation. Finally, changes in sCr can sometimes lag beyond the 48 h time period defined by the KDIGO criteria, and therefore a deterioration of renal function might have occurred following hospital discharge. This could have led to an underestimation of AKI incidence in our study. As a single value of eGFR does not define CKD, the true incidence of CKD could not be assessed. The definition of AKI is based on either sCr criteria or urine output criteria. We relied only on sCr criteria, which can be acutely elevated due to other factors, thus the diagnosis of AKI may have been overestimated. Patients with higher CPK were more likely to have lower eGFR. As information regarding patients’ weight was lacking, the relation of sCr to muscle mass could not be assessed. As STEMI patients treated conservatively were not included, the true effect of PCI on AKI could not be assessed.

### 4.2. Conclusions and Clinical Implications

Total serum CPK level elevations independently predict a higher risk for AKI in STEMI patients. As opposed to the setting of rhabdomyolysis in STEMI, a significantly increased risk for acute renal dysfunction has already been observed at relatively modest CPK level elevations. Higher CPK levels suggest a higher degree of myocardial damage, which may have resulted in acutely worse LV function, and more severe adverse hemodynamic effects resulting in higher risk for AKI. In the present study, we tried differentiating whether higher CPK levels directly (myoglobin-related kidney damage) or indirectly (through hemodynamic effects) affect kidney function by adjusting the risk for AKI in the multivariate models with LVEF and troponin levels measured during hospitalization. Although presumably contributing independently, it remains unclear whether direct myoglobin-related kidney damage is the main mechanism relating CPK levels with the risk for AKI.

Our findings may bear some clinical implications. As the treatment of established AKI in STEMI patients is often limited, focus should be on identifying patients with higher risk for deterioration of renal function. Withholding nephrotoxic drugs, liberal fluid resuscitation, or alkalizing patients’ urine may all be useful for patients at higher risk of AKI, identified amongst others by elevated CPK levels.

## Figures and Tables

**Table 1:** Baseline characteristics.

	Mild (CPK < 1000 U/L) *n* = 1603	Moderate (CPK = 1000–5000 U/L) *n* = 1111	Severe (CPK > 5000 U/L) *n* = 80	*p* Value
Age (years), mean ± SD	63 ± 13	61 ± 13	59 ± 13	<0.001
Gender (male), *n* (%)	1273 (79)	928 (84)	72 (90)	0.003
Hypertension, *n* (%)	773 (48)	447 (40)	28 (35)	<0.001
Diabetes mellitus, *n* (%)	440 (27)	221 (20)	10 (13)	<0.001
Family history of CAD, *n* (%)	340 (21)	252 (23)	21 (26)	0.42
Past AMI, *n* (%)	264 (17)	134 (12)	5 (6)	<0.001
Smoking, *n* (%)	767 (48)	593 (53)	37 (46)	0.01
Hyperlipidemia, *n* (%)	833 (52)	517 (47)	24 (30)	<0.001
Peak troponin (ng/L), mean ± SD	8938 ± 31,958	37,911 ± 108,427	70,779 ± 152,259	<0.001
LVEF (%), mean ± SD	48 ± 8	45 ± 7	40 ± 8	<0.001
LVEF ≤ 45%, *n* (%)	651 (41)	683 (63)	70 (88)	<0.001

CPK, creatine phosphokinase; SD, standard deviation; CAD, coronary artery disease; AMI, acute myocardial infarction; LVEF, left ventricular ejection fraction.

**Table 2:** Baseline and in-hospital renal outcomes.

	Mild (CPK < 1000 U/L) *n* = 1603	Moderate (CPK = 1000–5000 U/L) *n* = 1111	Severe (CPK > 5000 U/L) *n* = 80	*p* Value
Baseline eGFR ≤ 60 mL/min/1.73 m^2^, *n* (%)	388 (24)	236 (21)	28 (35)	0.009
Baseline eGFR (mL/minute/1.73 m^2^), mean ± SD	76 ± 25	77 ± 24	70 ± 19	0.01
Admission creatinine (mg/dL), mean ± SD	1.11 ± 0.5	1.10 ± 0.3	1.22 ± 0.3	0.05
Acute kidney injury, *n* (%)	125 (7.8)	122 (11)	21 (26)	<0.001
Creatinine change (mg/dL), mean ± SD	0.09 ± 0.27	0.13 ± 0.42	0.39 ± 0.81	<0.001
Peak creatinine (mg/dL), mean ± SD	1.20 ± 0.6	1.23 ± 0.6	1.64 ± 1.0	0.001
Serum NGAL levels (ng/mL), mean ± SD	90 ± 36	112 ± 40	183 ± 60	<0.001

CPK, creatine phosphokinase; eGFR, estimated glomerular filtration rate; NGAL, neutrophil gelatinase associated lipocalin.

**Table 3:** Multivariate binary logistic regression models predicting acute kidney injury.

	Model 1	*p* Value	Model 2	*p* Value
	OR (95% CI)		OR (95% CI)	
Gender (female)	1.2 (0.8–1.8)	0.5	1.1 (0.8–1.7)	0.5
Age (years)	1.01 (0.99–1.03)	0.3	1.01 (0.99–1.03)	0.2
Hypertension	1.9 (1.3–2.7)	0.001	1.8 (1.3–2.7)	0.001
LVEF (%)	0.93 (0.92–0.95)	<0.001	0.93 (0.91–0.95)	<0.001
eGFR (mL/minute/1.73 m^2^)	0.97 (0.96–0.98)	<0.001	0.97 (0.96–0.98)	<0.001
Diabetes mellitus	1.3 (0.9–1.8)	0.2	1.3 (0.9–1.8)	0.2
Hyperlipidemia	1.03 (0.7–1.4)	0.9	1.07 (0.8–1.5)	0.7
Family history of CAD	0.8 (0.5–1.4)	0.5	0.8 (0.5–1.4)	0.4
Smoking history	0.8 (0.6–1.2)	0.3	0.8 (0.6–1.2)	0.3
Past AMI	1.3 (0.8–1.9)	0.2	1.3 (0.8–1.9)	0.3
Peak troponin (ng/L)	1.0 (0.99–1.01)	0.8	1.0 (0.99–1.01)	0.8
CPK ≥ 1000 U/L	1.6 (1.1–2.2)	0.01		
CPK > 5000 U/L *			2.8 (1.4–5.6)	0.004

OR, odds ratio; CI, confidence interval; LV, left ventricular ejection fraction; eGFR, estimated glomerular filtration rate; CAD, coronary artery disease; AMI, acute myocardial infarction; CPK, creatine phosphokinase. * Eighty patients with CPK > 5000 U/L.
